# Variation of preoperative chest radiography utilization in Switzerland and its influencing factors: a multilevel study with claims data

**DOI:** 10.1038/s41598-018-35856-9

**Published:** 2018-11-30

**Authors:** Wenjia Wei, Oliver Gruebner, Viktor von Wyl, Beat Brüngger, Holger Dressel, Agne Ulyte, Eva Blozik, Caroline Bähler, Matthias Schwenkglenks

**Affiliations:** 1 Epidemiology, Biostatistics and Prevention Institute, University of Zurich, Zurich, Switzerland; 2 Department of Health Sciences, Helsana Insurance Group, Zurich, Switzerland; 3 Division of General Practice, University Medical Centre Freiburg, Freiburg, Germany

## Abstract

Clinical recommendations discourage routine use of preoperative chest radiography (POCR). However, there remains much uncertainty about its utilization, especially variation across small areas. We aimed to assess the variation of POCR use across small regions, and to explore its influencing factors. Patients undergoing inpatient surgery during 2013 to 2015 were identified from insurance claims data. Possible influencing factors of POCR included socio-demographics, health insurance choices, and clinical characteristics. We performed multilevel modelling with region and hospital as random effects. We calculated 80% interval odds ratios (IOR-80) to describe the effect of hospital type, and median odds ratios (MOR) to assess the degree of higher level variation. Utilization rates of POCR varied from 2.5% to 44.4% across regions. Higher age, intrathoracic pathology, and multi-morbidity were positively associated with the use of POCR. Female gender, choice of high franchise and supplementary hospital insurance showed a negative association. MOR was 1.25 and 1.69 for region and hospital levels, respectively. IOR-80s for hospital type were wide and covered the value of one. We observed substantial variation of POCR utilization across small regions in Switzerland. Even after controlling for multiple factors, variation across small regions and hospitals remained. Underlying mechanisms need to be studied further.

## Introduction

Preoperative chest radiography (POCR) is an example of a frequently overused healthcare service, discouraged by international clinical practice guidelines^1^. The Choosing Wisely initiative^2^ launched in the US called for more caution in the use of POCR for asymptomatic patients due to its potential uselessness, harm and cost^3^. POCR has been shown to have negligible influence on subsequent patient management as well as clinical outcomes, and to result in significant costs^4–7^. A Swiss version of the Choosing wisely – “Smarter Medicine” initiative was launched by the Society for General Internal Medicine (SGAIM) in May 2014^8^. Avoidance of POCR for asymptomatic patients is among the top five recommendations published by the SGAIM in May 2016 addressing the overuse of healthcare services^9^.

A recent study by Blozik *et al*. investigated the degree and geographic distribution of POCR utilization in Switzerland across large geographic units. Excessive use of POCR was undetectable in that study, but it demonstrated significant variation in the utilization rates (6–28%) at the cantonal level^10^. However, differences between smaller geographic regions were not explored. Summary measures such as national or cantonal POCR utilization rates may mask local trends and true distribution patterns. In Switzerland, there have been no cantonal policy or regulation regarding POCR which might be the driver of utilization variation, and we assume there could be significant within canton variation of POCR use in small regions. Analysis of variation of healthcare utilization in smaller geographic areas has considerable potential to support the planning and delivery of healthcare, through offering valuable insights to health professionals, health policymakers and the general public^11,12^.

Different factors (patient, provider, and region-specific characteristics) may affect POCR utilization, which has not been fully explored to date. One study on preoperative testing before low-risk surgical procedures in Canada showed that POCR utilization was associated with age, preoperative anaesthesia consultation, preoperative medical consultation and healthcare institution^13^. However, the authors did not take the potential impact of health insurance characteristics or patients’ residence into consideration.

The utilization of this potentially avoidable procedure across small areas in Switzerland remains uncertain. Building on the work by Blozik *et al*.^10^, which provided the first overview of POCR utilization in Switzerland, we proceeded to an explicit small area analysis of POCR utilization and variation. The aim of our study was to assess the variation of POCR utilization across 106 Spatial Mobility regions (MS regions), and to investigate the patient, hospital and regional factors potentially influencing POCR utilization and variation in Switzerland.

## Material and Methods

### Study population

We studied patients who received Swiss mandatory health insurance (Obligatorische Krankenpflegeversicherung, OKP) from the Helsana Group. Helsana is one of the largest health insurance companies in Switzerland, and the Helsana database underlying this study included mandatory health insurance claims from approximately 1.2 million people per year, covering about 15% of the whole Swiss population. The study population was patients enrolled with Helsana who were older than 18 years and underwent non-emergency inpatient surgery from 2013 to 2015. We excluded patients with incomplete coverage of mandatory health insurance during 2013 and 2014, asylum seekers, patients living outside Switzerland, Helsana employees, patients with incomplete address information, patients living in nursing homes with lump-sum reimbursement of medication, and emergency inpatient stays. Only the first hospitalization per person during the study period was considered. The data used in the present study was the same as that in the study by Blozik *et al*.^10^.

Basic health insurance bought from a private market of health insurance companies is mandatory for all Swiss residents. The insurance companies are obliged to offer mandatory health insurance at the same price to everyone regardless of their health status. Premiums are lower for children and young adults; they differ between geographic regions. All appropriate and cost-effective inpatient or outpatient medical treatments are covered by mandatory health insurance. Supplementary hospital insurance is optional and allows for hospitalization in a semiprivate or private ward and treatment in another canton^14^. Enrolees can choose between various annual deductible costs (i.e., a “franchise”) ranging from 300 to 2,500 Swiss Francs. The higher the franchise chosen, the lower the premium to pay. There are managed care and standard fee-for-service models of mandatory health insurance. Insured people selecting managed care models have to first consult a specific type of healthcare provider (i.e., a group practice, a defined family doctor, or a telemedicine centre)^14,15^. Thus insured people with managed care models pay fewer premiums compared to standard model users while they use the same fee-for-service tariff.

The study data provided by Helsana were anonymized. According to the national ethical and legal regulations, ethical approval was not needed for this type of analysis. This was confirmed by a waiver of the competent ethics committee (Kantonale Ethikkommission Zürich, waiver dated 11^th^ January 2017).

### Outcome and explanatory variables

The outcome variable was the performance of ambulatory chest radiography within two months before any inpatient surgery^16^. Inpatient surgeries were derived from the Swiss Diagnosis Related Groups (DRG) code. Possible influencing factors for POCR performance selected were based on the previous, similar literature^10,13^ or were persumed to be logic, they included:

1. Socio-demographic characteristics, including age, gender, language region (German, French or Italian), purchasing power index per household (describing the per capita income of a postal code region as a proxy for the socioeconomic status of the respective region), and urban or rural residence;
2. Health insurance characteristics, including insurance coverage: only mandatory health insurance or also supplementary health insurance, e.g. the supplementary hospital care insurance, high franchise (more than 500 Swiss Francs), and standard or managed care insurance models;
3. Type of hospital performing surgery. The four hospital types were central hospital (offering the highest level of healthcare services, including university hospitals), primary hospital, surgical hospital and other specialized clinic – as categorized by the Swiss Federal Statistical Office (SFSO);
4. Clinical characteristics, for instance, multi-morbidity, indication of intrathoracic pathology (patients with either cardiovascular disease or respiratory disease based on pharmaceutical cost groups). Since Swiss health insurance claims data do not have a meaningful degree of diagnostic data for outpatient services, pharmaceutical cost groups (PCG) are used to deduce chronic morbidity at the patient level based on drug use^17^. Multi-morbidity was defined as the presence of at least two PCGs.

### Geographic unit

Instead of the 26 Swiss cantons, we used 106 MS regions as the geographic units for small area analysis of POCR utilization and variation. MS regions are defined by the SFSO and used in particular as a microregional intermediate level for numerous scientific and regional policy purposes. They are characterized by a certain spatial homogeneity and obey the principle of small-scale labor market areas^18^. Each patient’s residence was assigned to the corresponding MS region in the claims data.

### Statistical analysis

First, we performed a descriptive analysis of the eligible patients’ characteristics, including their socio-demographic, insurance, clinical and hospital characteristics. Second, to have an intuitive, visual impression of the detailed distribution of POCR utilization in Switzerland, we aggregated the patient level outcome and explanatory variables at the MS regional level. Specifically, for each MS region, we calculated the POCR rate, mean age, percentage of women, mean purchasing power index per household, percentage of patients with high franchise and with standard fee-for-service model in the mandatory health insurance, with only mandatory insurance, with supplementary hospital care insurance, with indication of intrathoracic pathology, with multi-morbidity. We also assessed the percentage of patients receiving surgery in each hospital type. We then mapped all relevant variables using the Geographic Information System (GIS) software package QGIS (version 2.14.16)^19^ to show their geographic distribution. Third, to explore the spatial autocorrelation present in these variables, we computed the Moran’s I statistic and calculated Local Indicators of Spatial Association (LISA) using GeoDa (version 1.10)^20^ that were subsequently mapped with GIS. Moran’s I measures the correlation of a variable with itself through space, it ranges from −1 to 1. If the value of Moran’s I is zero or very close to 0 (p > 0.05), it suggests there is no spatial autocorrelation (null hypothesis: the variable is totally randomly distributed through space). If Moran’s I is positive (p < 0.05), indicating there is positive spatial autocorrelation, namely the variable of one region is more similar to the regions close to it compared to regions far from it, and the vice versa if Moran’s I is negative. LISA shows exactly where the significant spatial clustering or dispersion happens locally.

To investigate the factors that potentially affected the utilization of POCR, we first conducted logistic regression at the patient level to describe the associations between use of POCR and all potential predictors other than the geographic unit of residence. We applied a manual, step-by-step variable selection process to develop a multivariable logistic regression model with only the relevant variables (with a significant coefficient, p < 0.05). This multivariable model was then checked for multicollinearity and tested for goodness of fit with the receiver operating characteristic (ROC) curve. We calculated the mean residuals per MS region and checked the spatial correlation with Moran’s I statistic.

The nesting of all individuals within MS regions implied a hierarchical data structure. In order to take this into account, we additionally performed multilevel logistic regression (multilevel model 1) with patients as the 1^st^ level and MS regions as the 2^nd^ level. Besides, we also considered the hospitals where surgeries were performed as a random effect in multilevel modeling. However, the 3-level data structure (patient – hospital – MS region of residence) was not entirely hierarchical, namely not all patients residing in one MS region had surgeries in hospitals within the same MS region. To solve this cross-classification issue, we further built a cross-classified multilevel model (multilevel model 2) taking both MS regions and hospitals into consideration as random effects. As the cluster-level covariate in multilevel model 2, the effect of hospital type was quantified using the 80% interval odds ratio (IOR-80)^21–23^. This decision was taken because other than individual-level covariates in multilevel models, cluster-level covariates take only one value in each cluster. The interpretation of standard odds ratios is hence not straightforward for cluster-level covariates. Considering the distribution of odds ratios comparing two patients with different cluster-level covariate values (having surgeries in hospitals of a different type), but identical values for all other covariates, the IOR-80 covers the middle 80% of such odds ratios and has been recommended to describe cluster-level associations. The IOR-80 is narrow if between-cluster variation is small, and vice versa. If IOR-80 contains the value of one, the between-cluster variation is more important than the effect of the cluster-level covariate, if not, the latter is more relevant. To estimate the degree of the random variation, we calculated the median odds ratio (MOR) for both multilevel models. The MOR compares the adjusted odds of POCR utilization in two patients with the same covariates except residing in two randomly selected MS regions (or having surgery by two randomly selected hospitals), and it can be interpreted as the median of these ORs. MOR is always above or equal to one since it is the median odds ratio between the person with a higher propensity and the person with a lower propensity for the outcome of interest^21–23^. MOR could be used directly for comparison with ORs of fixed-effect variables^21–23^. We then drew caterpillar plots of higher-level residuals to identify the MS regions that were significantly different from the average of all MS regions. At last, we checked spatial correlation of the two multilevel models’ residuals at MS region level using Moran’s I statistic. Due to the multilevel nature of data and the potential effect of MS region and hospitals on POCR utilization, we regarded the cross-classified multilevel regression model as our main model. To justify the random effects, we also calculated the variation partition coefficient (VPC) for both the MS region and hospital levels, in a cross-classified model without covariates.

## Results

In total, 47,215 insured patients who experienced hospitalization for non-emergency surgery were analyzed in our study. Among them, 6,121 (13.0%) had ambulatory chest radiography within two months before surgery. Table 1 shows the characteristics of all included patients, patients with POCR, and patients without POCR, respectively. Women accounted for 57.4% of the total study population, and the mean age was 60.3 years. Compared to patients without POCR, patients with POCR were older (mean age: 68.4 vs. 59.1 years old), more frequently male and wealthier. They also preferred mandatory plus additional health insurance, high franchise, standard insurance model and supplementary hospital care insurance; and they were more likely to have an intrathoracic pathology and multi-morbidity; finally, they more often had surgery in a primary hospital or surgical hospital.

**Table 1:** Characteristics of 47215 insured patients undergoing inpatient surgery during the year 2013 to 2015.

Characteristics	Total	Without POCR	With POCR
n	47215	41094 (87.0%)	6121 (13.0%)
Female	27086 (57.4%)	23829 (58.0%)	3257 (53.2%)
Age (mean, SD)	60.3 (17.2)	59.1 (17.4)	68.4 (12.6)
Purchasing power index per household	101.7 (22.7)	101.6 (22.4)	102.8 (24.3)
Urban residence	36457 (77.2%)	31783 (77.3%)	4674 (76.4%)
Language region			
German	37547 (79.5%)	32615 (79.4%)	4932 (80.6%)
French	6157 (13.0%)	5457 (13.3%)	700 (11.4%)
Italian	3511 (7.4%)	3022 (7.4%)	489 (8.0%)
Intrathoracic pathology indication^a^	24566 (52.0%)	20479 (49.8%)	4087 (66.8%)
Multi-morbidity^b^	26267 (55.6%)	22056 (53.7%)	4211 (68.8%)
Insurance coverage			
Mandatory	10875 (23.0%)	9674 (23.5%)	1228 (20.1%)
Mandatory and supplementary	36340 (77.0%)	31447 (76.5%)	4893 (79.9%)
Supplementary hospital care insurance	11858 (25.1%)	10153 (24.7%)	1705 (27.9%)
High franchise (>500 Swiss Francs)	7799 (16.5%)	7163 (17.4%)	636 (10.4%)
Mandatory insurance models			
Standard	24108 (51.1%)	20742 (50.5%)	3366 (55.0%)
Managed care	23107 (48.9%)	20352 (49.5%)	2755 (45.0%)
Type of hospital performing surgery^c^			
Central hospital	19711 (41.7%)	17511 (42.6%)	2200 (35.9%)
Primary hospital	21269 (45.0%)	18298 (44.5%)	2971 (48.5%)
Surgical hospital	5130 (10.9%)	4317 (10.5%)	813 (13.3%)
Other specialized clinic	1105 (2.3%)	968 (2.4%)	137 (2.2%)

POCR: preoperative chest radiography; SD: standard deviation. ^a^Patients with either cardiovascular disease or respiratory disease based on pharmaceutical cost groups (PCG); ^b^Patients with two or more than two chronic diseases based on PCG; ^c^Categorized according to the Swiss Federal Statistical Office (SFSO).

**Figure 1:**
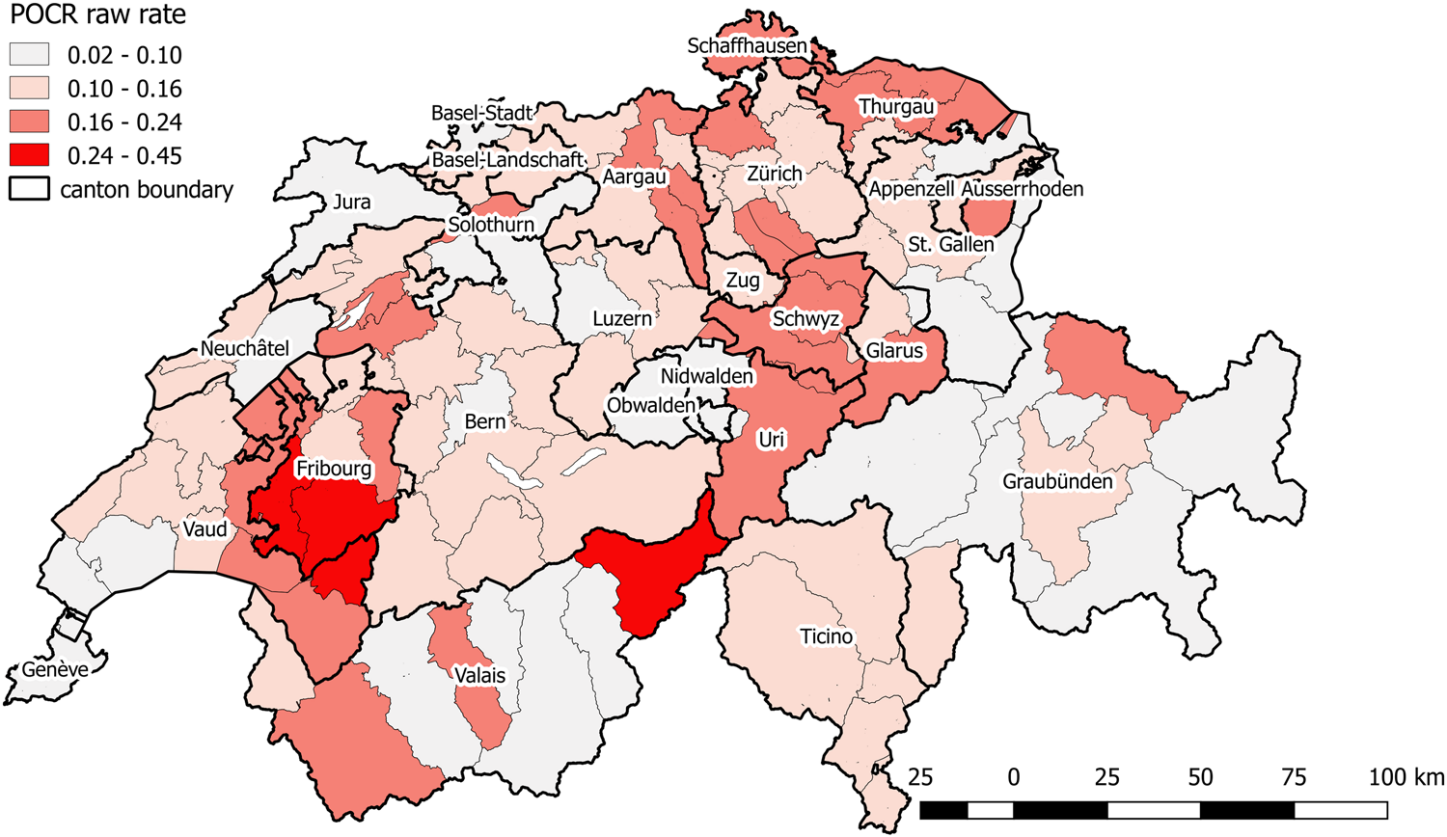
Geographic distribution of POCR utilization across MS regions.

**Figure 2:**
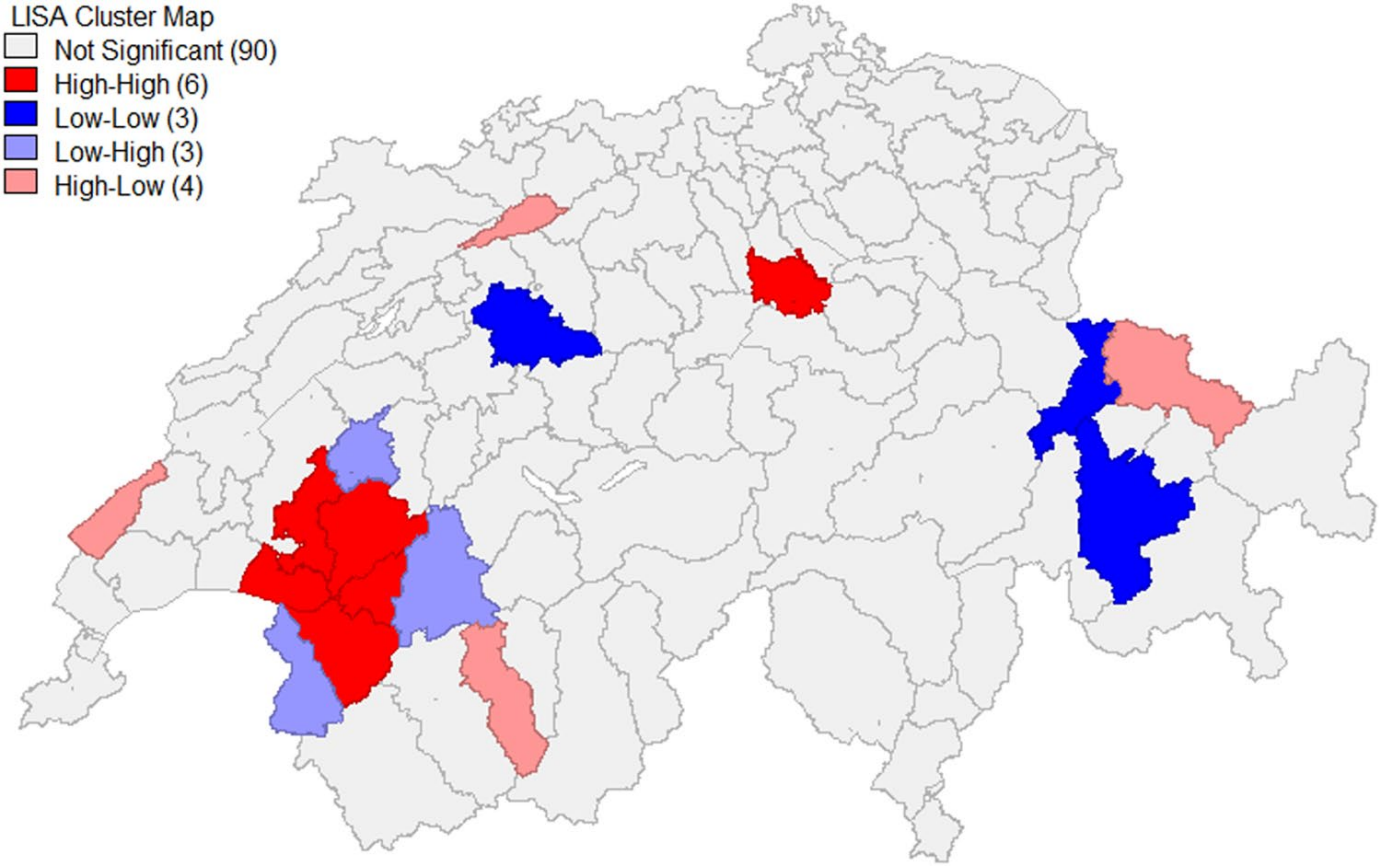
LISA cluster map of POCR raw rates across MS regions.

POCR raw rates varied from 2.5% to 44.4% across 106 MS regions (the range was 2.3% to 30.7% after age standardization). Geographic distribution of POCR utilization across MS regions is shown in Fig. 1. There were considerable geographic variation and clustering of POCR rates. Geographic distribution of all considered influencing factors are shown in Supplementary Fig. S1. Moran’s I value of POCR raw rates across MS regions was 0.26 and was statistically significant (*p* < 0.001). It indicates substantial spatial autocorrelation in POCR utilization, namely the POCR use is not randomly distributed among MS regions, and the POCR rate of one region is more similar to its neighbouring regions compared to regions far away. Figure 2 presents a LISA cluster map of POCR raw rates with several significant clusters of POCR utilization across Switzerland. The main high-high spatial cluster (regions with high POCR rates surrounded by neighbours also with high rates) was detected around the canton of Fribourg. The Moran’s I statistic and LISA clustering maps of possible influencing factors are shown in Supplementary Fig. S2.

In the logistic regression model (Table 2), higher age, indication of intrathoracic pathology, multi-morbidity, higher purchasing power index per household, and receiving surgery in hospitals providing lower levels of care (i.e., primary hospitals, surgical hospitals and other specialized clinics) were positively associated with the use of POCR. In contrast, female gender, urban residence, living in the French-speaking compared to German-speaking region, choice of an insurance model with high deductibles and supplementary hospital care insurance showed a negative association. We did not find multi-collinearity and the area under ROC curve (AUC) was 0.67. There was no significant effect modification identified in the model. Moran’s I of mean model residuals per MS region was 0.28 (p < 0.01), indicating the presence of residual spatial correlation remained after the modeling of covariate effects. Therefore, the model assumption of independent residuals was not perfectly met and the model needs to be improved further.

**Table 2:** Results of logistic regression model and multilevel models for the association between POCR utilization and influencing factors.

	Logistic regression	Multilevel model 1^d^	Multilevel model 2^e^
Fixed effects (OR and 95% CI)			
Age	1.033 (1.031, 1.036)	1.034 (1.031, 1.036)	1.034 (1.032, 1.036)
Female gender	0.841 (0.796, 0.890)	0.838 (0.793, 0.887)	0.840 (0.794, 0.890)
High franchise (>500 Swiss Francs)	0.756 (0.690, 0.829)	0.755 (0.688, 0.828)	0.746 (0.679, 0.818)
Supplementary hospital care insurance	0.934 (0.876, 0.995)	0.928 (0.870, 0.989)	0.901 (0.842, 0.965)
Intrathoracic pathology indication^a^	1.137 (1.055, 1.225)	1.145 (1.062, 1.235)	1.149 (1.064, 1.240)
Multi-morbidity^b^	1.107 (1.026, 1.193)	1.113 (1.031, 1.120)	1.122 (1.039, 1.211)
Purchasing power index per household	1.002 (1.001, 1.004)		
Urban residence	0.911 (0.852, 0.973)		
Language region			
German	1		
French	0.852 (0.782, 0.929)		
Italian	1.059 (0.954, 1.177)		
Type of hospital performing surgery^c^			
Central hospital	1	1	1
Primary hospital	1.356 (1.276, 1.441)	1.367 (1.281, 1.458)	1.210 (0.932, 1.571)
* IOR-80*			0.45–3.26
Surgical hospital	1.571 (1.436, 1.718)	1.621 (1.478, 1.778)	1.436 (1.036, 1.991)
* IOR-80*			0.53–3.88
Other specialized clinic	1.212 (1.002, 1.465)	1.291 (1.064, 1.566)	1.434 (0.917, 2.240)
* IOR-80*			0.53–3.87
Random effects			
MOR_MS_		1.49	1.25
MOR_HP_			1.69
Moran’s I of residuals	0.29 (p < 0.01)	0.34 (p < 0.01)	0.066 (p = 0.115)

OR: odds ratio; CI: confidence interval; MOR_MS_: median odds ratio of MS region effect; MOR_HP_: median odds ratio of hospital effect; IOR-80: 80% interval odds ratio. ^a^Patients with either cardiovascular disease or respiratory disease based on pharmaceutical cost groups (PCG); ^b^Patients with two or more chronic diseases based on PCG; ^c^Categorized according to the Swiss Federal Statistical Office (SFSO); ^d^Estimating random effects for MS regions only; ^e^Cross-classified model estimating random effects for both MS regions and hospitals.

**Figure 3:**
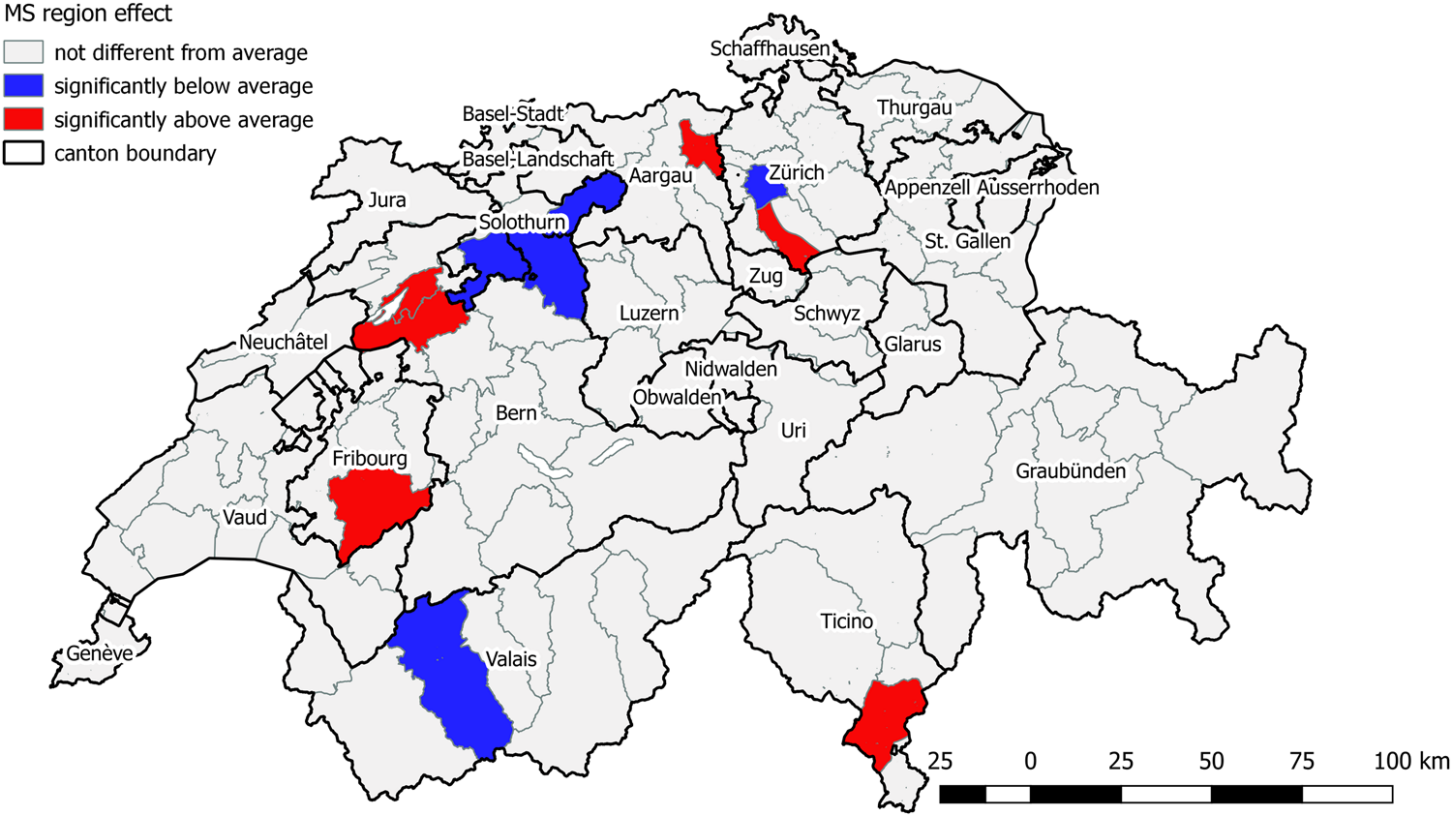
MS regions significantly different from the average MS region effect identified from caterpillar plot of the cross-classified multilevel model.

Indicators of purchasing power index per household, urban residence, and language region were not significant and therefore excluded from both multilevel models (Table 2). The effect of hospital type on POCR utilization in multilevel model 1 was remarkable, with odds ratios of 1.37 (95% CI: 1.28–1.46) for primary hospital, 1.62 (95% CI: 1.48–1.78) for surgical hospitals and 1.29 (95% CI: 1.06–1.57) for other specialized clinics compared to central hospitals. In multilevel model 2, only the category of surgical hospitals had a significant OR of 1.44 (95% CI: 1.04–1.99) compared to central hospitals, while the joint p-value for the overall hospital type variable was 0.137. Consistent with that, the IOR-80 for each hospital type compared to central hospitals was relatively wide and contained the value of one, reflecting substantial unexplained variation between hospitals and implying that hospital type did not account for much of this heterogeneity. The median odds ratio (MOR) of MS region in multilevel model 1 was 1.49, suggesting a large amount of variation between MS regions. In multilevel model 2, the MOR of MS region (MOR_MS_) decreased to 1.25, indicating only moderate heterogeneity between MS regions, while the MOR of hospital was higher (MOR_HP_ = 1.69), suggesting a large amount of variation between hospitals, which was also reflected in the wide IOR-80. From the caterpillar plot of multilevel model 2, we identified 11 MS regions significantly differing from the average MS region random effect. Among them, 5 MS regions had a significantly lower probability of performing POCR compared to the average probability (one MS region in canton Zurich, one in canton Bern, one in canton Valais and two in canton Solothurn), and 6 had a significantly higher probability (one in canton Zurich, one in canton Fribourg, one in canton Aargau, one in canton Ticino and two in canton Bern). Figure 3 shows the geographic locations of these 11 MS regions.

The Moran’s I of multilevel model 1 residuals at MS region level was 0.34 (p < 0.01). However, after taking hospital into consideration as random effect, multilevel model 2 residuals at MS region level showed little spatial correlation (Moran’s I = 0.066, p = 0.115), implying that the model assumption was met and cross-classified multilevel model solved spatial correlation issue well. The VPCs for the MS region and hospital levels were 1.6% and 8%, respectively.

## Discussion

We found substantial variation in POCR utilization rates across 106 MS regions in Switzerland. Different factors including patient socio-demographic and clinical characteristics, health insurance features, and hospital-related factors appeared to affect POCR utilization. Considerable variation of POCR utilization across MS regions and especially hospitals persisted even after the adjustment for these factors, hinting at the existence of additional influences not covered by our dataset.

Due to very limited clinical and economic benefit of POCR in asymptomatic patients, both the US Choosing Wisely initiative and Smarter Medicine in Switzerland have put POCR on their lists of procedures that should be avoided except for special situations^3,9^. A study from the US showed a prevalence of 91.5% for POCR among patients with unremarkable history and physical examination results in 2013^24^. Another US study using 2009 Medicare claims data showed a 5.5% POCR utilization rate^25^. Two studies in Canada examined hospital databases from 2005 to 2007 and 2008 to 2013, and reported 23.3% and 10.8% POCR utilization rates in Alberta and Ontario, respectively^13,26^. Overall POCR rate in our study was 13.0%, which did not differ much from the previous findings except the one for the US with a 91.5% POCR rate (for which we have not found an obvious explanation). However, these results may not be entirely comparable because of different sample selection and outcome definition. For example, there were differences regarding the databases used (health insurance claims data, further healthcare administrative data or hospital discharge data), the age ranges of the study populations (patients above 18 years or only the elderly patients), the surgery types included (inpatient vs. outpatient surgeries, low risk surgeries, elective surgeries, or non-cardiothoracic surgeries), and the time period before surgery (“preoperative” was defined inconsistently as 14, 30 or 60 days before surgery in different studies). In our study, we considered POCR performed within two months before any inpatient surgeries in all patients, without excluding cardiothoracic surgeries or patients with cardiopulmonary diseases. Therefore, a certain degree of POCR utilization was expected and would be justified in the present study.

The first study investigating the geographic variation of POCR utilization in Switzerland so far, by Blozik *et al*., demonstrated a substantial variation of POCR rates at the cantonal level. Across the 26 Swiss cantons, the observed minimum was 6% in the canton of Obwalden and the maximum 28% in the canton of Schwyz^10^. When using smaller geographic units – MS regions in our study, we observed considerable small area variation (raw rate of POCR utilization rate across MS regions: 2.5% to 44.4%), also within cantons. The three MS regions with the highest POCR rates were in cantons of Valais and Fribourg, and the three with the lowest POCR rates were in cantons of St. Gallen, Valais and Graubünden.

Our cross-classified multilevel results suggested that the most relevant factors of POCR utilization available in our claims data were older age, male gender, indication of intrathoracic pathology, choice of an insurance model with low deductibles, having supplementary hospital insurance, and multi-morbidity. Older patients generally have worse health status and more comorbidities, thus they tend to be treated with more caution; the same applies to patients with multi-morbidity. Lower use of POCR in women may be partly related to different types of surgery performed on men and women; this possibility could be further explored with detailed surgery information. Patients with cardiovascular or respiratory disease were more likely to receive POCR, not unexpected for patients with an intrathoracic pathology. The health insurance related factors indicated that patients choosing a higher franchise had a lower probability of POCR. One reason may be that patients choosing a higher franchise are normally healthier, besides, higher out-of-pocket costs could make them more reluctant to undergo POCR. Patients with supplementary hospital care insurance had a slightly lower probability of POCR. This finding might be due to patients’ selection of “better” or “more expensive” care such as ultrasound or MRI, compared to POCR. However, we expect inpatient POCR to be generally rare (see below). There have been few other studies investigating possible influencing factors of POCR utilization. One US study^13^ found that older age, certain comorbidities and preoperative consultations played an important role. Our finding of an impact of health insurance-related factors as an example of non-clinical patient-sided factors on POCR is relatively novel.

The MOR_MS_ in multilevel model 2 implies that moderate unexplained variation of POCR utilization across MS regions persisted after controlling for the available influencing factors. Based on both the wide IOR-80 of hospital type and the relatively high MOR_HP_ value in multilevel model 2, the between hospital variation of POCR utilization was substantial and cannot be explained by hospital type. Hospitals made a more relevant contribution than MS regions to the variation of POCR utilization. Similarly, Blozik *et al*. also observed large variance between hospitals within a canton, and concluded that individual hospitals proceed very differently with the placement of the POCR^10^. The residual between-MS region and between-hospital variation after modeling might be due to certain regional or hospital-level determinants that we could not control for in our study, for instance, provider density, attitude of physicians or patients, acceptance of guidelines. Although there was very few literature studying impact factors of POCR variation, some studies exploring factors influencing utilization of other health services might give us some insight into possible neglected predictors. For example, Chen I *et al*. concluded that neighbourhood education could affect hysterectomy utilization rate^27^. Another study found that primary care use was influenced by the density of primary care practices^28^. They might be included in further studies. In addition, the underlying mechanisms that account for the 11 MS regions being significantly different from the average effect should also be further investigated closely and locally for better health service provision and resource allocation. Most previous studies^29–31^ only conducted descriptive assessments of regional variations of healthcare utilization, reporting, for example, interquartile range, extremal quotient (EQ), coefficient of variance (CV) and systematic component of variation (SCV). They usually did not control for potential influencing factors. Our study highlighted a more advanced and comprehensive method of regional variation estimation through multilevel modelling, which we will transfer and apply to studies planned for other healthcare services of interest.

The present study was based on claims data before the “Smarter Medicine” initiative was introduced in Switzerland in 2016. A possible follow-up study might provide additional insights into the influence of negative recommendation on POCR utilization. The study had a few limitations. First, due to the limitation of health insurance claims data some potentially important variables such as whether or not there was an indication for POCR, the physicians’ and patients’ preferences, etc. were lacking. Also, our data on POCR utilization were based on claims data from the outpatient sector. We had no details of services, treatments or procedures during inpatient episodes. POCR use during inpatient stays would not have been captured. However, due to financial incentives encouraging the transfer of diagnostic measures to before inpatient stays, we assume that inpatient POCR occurred relatively rarely^10^. We did not have information on where the outpatient POCR were performed, but we do not consider this as very relevant for the decision for or against POCR use. Furthermore, the results came from a single health insurance company in Switzerland. Enrolees of other Swiss health insurers might theoretically show different patterns of use. However, the results presented here were based on an insured population of 1.2 million people from all regions of Switzerland. Helsana internal data show no evidence of deviation in basic characteristics of its own customers compared to the whole population. The benefit package of the obligatory health insurance is defined at the federal level and the same for all health insurance companies, and all physicians collaborate with all insurance providers. Thus we assume no huge difference between our study population from Helsana and whole Swiss population. Even if the Helsana population is not perfectly representative of whole population, we believe it has no big impact on the association results in our study. The results should be generalizable to a large extent for the whole Switzerland. In addition, theoretically, there might have been a surgery because of the chest radiography which we were not able to identify, although we believe the proportion of such situation would be quite small.

In conclusion, our study observed substantial variation of POCR utilization across MS regions in Switzerland. Patients’ socio-demographics and clinical characteristics, choice of health insurance, and hospital-related factors influenced POCR utilization. Despite controlling for these influencing factors, variation across MS regions and especially across hospitals persisted, implying a hospital specific effect. Underlying mechanisms need to be further clarified.

## Electronic supplementary material

Supplementary figures

## Data Availability

The dataset that supports the findings of the current study are from the Helsana Group, but are not publicly available as they are individual-level, health-related claims data on human subjects, albeit anonymised. However, the data are available from the Helsana Group, upon reasonable request.

## References

[1] In *National Guideline Centre. Preoperative Tests (Update): Routine Preoperative Tests for Elective Surgery* (National Institute for Health and Care Excellence (UK). Copyright (c) National Institute for Health and Care Excellence 2016., 2016). 27077168

[2] Cassel CK, Guest JA (2012). Choosing wisely: helping physicians and patients make smart decisions about their care. Jama.

[3] Choosing Wisely Initiative. Chest X-rays Before Surgery (2012).

[4] Chung F, Yuan H, Yin L, Vairavanathan S, Wong DT (2009). Elimination of preoperative testing in ambulatory surgery. Anesthesia and analgesia.

[5] Cree, M. L. D. Routine Preoperative Tests – Are They Necessary?, (Institute of Health Economics, 2007).

[6] Fritsch G (2012). Abnormal pre-operative tests, pathologic findings of medical history, and their predictive value for perioperative complications. Acta anaesthesiologica Scandinavica.

[7] Rao VM, Levin DC (2012). The overuse of diagnostic imaging and the Choosing Wisely initiative. Annals of internal medicine.

[8] Gaspoz JM (2015). Smarter medicine: do physicians need political pressure to eliminate useless interventions?. Swiss medical weekly.

[9] SSGIM. *Top Five List for ≪Choosing Wisely≫ in the Inpatient Sector Published*., http://www.smartermedicine.ch/de/top-5-listen/stationaere-allgemeine-innere-medizin.html (2016).

[10] Blozik E, Brungger B, Reich O (2017). Medical Overuse in Switzerland: How Frequent is Preoperative Chest Radiography?. Praxis.

[11] Kephart, G. A. Y. *et al*. Small area variation in rates of high-cost healthcare use across Nova Scotia. (Maritime SPOR SUPPORT Unit. Halifax, Nova Scotia) (2016).

[12] Luft HS (2012). From small area variations to accountable care organizations: how health services research can inform policy. Annual review of public health.

[13] Kirkham KR (2015). Preoperative testing before low-risk surgical procedures. CMAJ: Canadian Medical Association journal = journal de l’Association medicale canadienne.

[14] Blozik E, Signorell A, Reich O (2016). How does hospitalization affect continuity of drug therapy: an exploratory study. Therapeutics and clinical risk management.

[15] Reich O, Rapold R, Flatscher-Thoni M (2012). An empirical investigation of the efficiency effects of integrated care models in Switzerland. International journal of integrated care.

[16] Bugar JM, Ghali WA, Lemaire JB, Quan H (2002). Utilization of a preoperative assessment clinic in a tertiary care centre. Clinical and investigative medicine. Medecine clinique et experimentale.

[17] Huber CA, Szucs TD, Rapold R, Reich O (2013). Identifying patients with chronic conditions using pharmacy data in Switzerland: an updated mapping approach to the classification of medications. BMC public health.

[18] *Bundesamt für Statistik. MS-Regionen*, https://www.bfs.admin.ch/bfs/de/home/statistiken/raum-umwelt/nomenklaturen/msreg.assetdetail.415729.html (2016).

[19] QGIS Development Team QGIS Geographic Information System. *Open Source Geospatial Foundation Project*, http://qgis.osgeo.org (2017).

[20] Anselin L, Syabri I, Kho Y (2006). GeoDa: An Introduction to Spatial Data Analysis. Geographical Analysis.

[21] Larsen K, Merlo J (2005). Appropriate assessment of neighborhood effects on individual health: integrating random and fixed effects in multilevel logistic regression. American journal of epidemiology.

[22] Larsen K, Petersen JH, Budtz-Jorgensen E, Endahl L (2000). Interpreting parameters in the logistic regression model with random effects. Biometrics.

[23] Ohlsson H (2005). Understanding adherence to official guidelines on statin prescribing in primary health care–a multi-level methodological approach. European journal of clinical pharmacology.

[24] Rosenberg A (2015). Early Trends Among Seven Recommendations From the Choosing Wisely Campaign. JAMA internal medicine.

[25] Schwartz AL, Landon BE, Elshaug AG, Chernew ME, McWilliams JM (2014). Measuring low-value care in Medicare. JAMA internal medicine.

[26] Thanh NX, Rashiq S, Jonsson E (2010). Routine preoperative electrocardiogram and chest x-ray prior to elective surgery in Alberta, Canada. Canadian journal of anaesthesia = Journal canadien d’anesthesie.

[27] Chen I (2017). Social and Geographic Determinants of Hysterectomy in Ontario: A Population-Based Retrospective Cross-Sectional Analysis. Journal of obstetrics and gynaecology Canada: JOGC = Journal d’obstetrique et gynecologie du Canada: JOGC.

[28] Briggs LW, Rohrer JE, Ludke RL, Hilsenrath PE, Phillips KT (1995). Geographic variation in primary care visits in Iowa. Health services research.

[29] Carlisle DM, Valdez RB, Shapiro MF, Brook RH (1995). Geographic variation in rates of selected surgical procedures within Los Angeles County. Health services research.

[30] McPherson K, Strong PM, Epstein A, Jones L (1981). Regional variations in the use of common surgical procedures: within and between England and Wales, Canada and the United States of America. Social science & medicine. Part A, Medical sociology.

[31] OECD. *Geographic Variations in Health Care*. (OECD Publishing).

