# Supplementary material for: Variation of preoperative chest radiography utilization in Switzerland and its influencing factors: a multilevel study with claims data
Source: Sci Rep. 2018 Nov 30;8:17475. doi: 10.1038/s41598-018-35856-9 (PMC6269528; doi:10.1038/s41598-018-35856-9)

Variation of preoperative chest radiography utilization in Switzerland and its influencing factors: a multilevel study with claims data

Wenjia Wei^1^*, Oliver Gruebner^1^, Viktor von Wyl^1^, Beat Brüngger^2^, Holger Dressel^1^, Agne Ulyte^1^, Eva Blozik^2, 3^, Caroline Bähler^2^, Matthias Schwenkglenks^1^

^1^Epidemiology, Biostatistics and Prevention Institute, University of Zurich, ^2^Helsana Group, Department of Health Sciences, ^3^Division of General Practice, University Medical Centre Freiburg, Freiburg, Germany

*Correspondence to [wenjia.wei@uzh.ch]

Supplementary Figure S1. Geographic distribution of possible influencing factors of preoperative chest radiography (POCR) use in Switzerland.
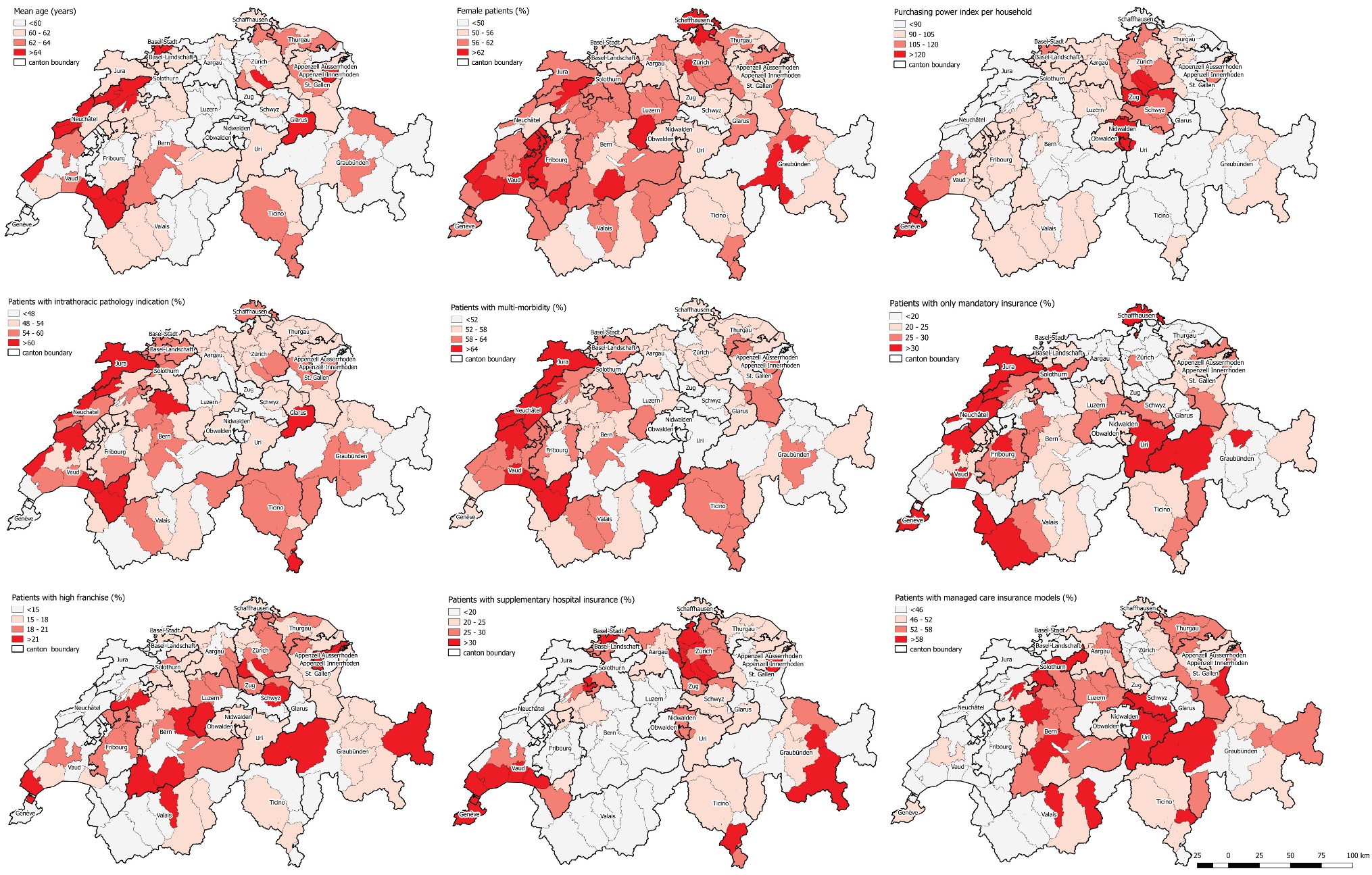


Supplementary Figure S2. Local Indicators of Spatial Association (LISA) clustering maps of possible influencing factors of preoperative chest radiography (POCR) use in Switzerland.


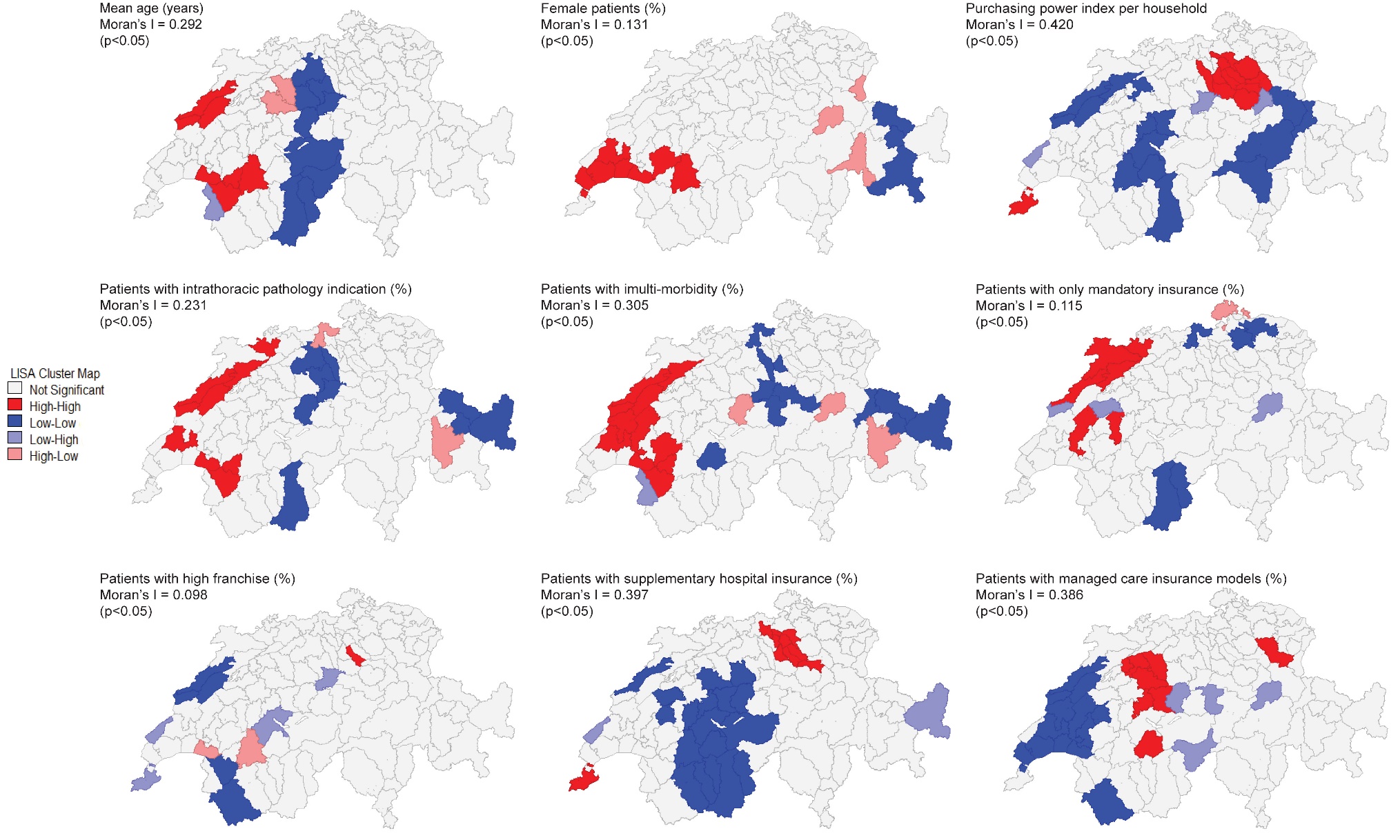

Supplement: Supplementary file 1 — Supplementary figures [file 41598_2018_35856_MOESM1_ESM.docx]
